# Meta-Analysis of Transcriptional Responses to Mastitis-Causing *Escherichia coli*

**DOI:** 10.1371/journal.pone.0148562

**Published:** 2016-03-02

**Authors:** Sidra Younis, Qamar Javed, Miroslav Blumenberg

**Affiliations:** 1 The R. O. Perelman Department of Dermatology, Department of Biochemistry and Molecular Pharmacology, NYU Langone Medical Center, New York, United States of America; 2 Department of Biochemistry, Quaid-i-Azam University, Islamabad, Pakistan; Ella Foundation, INDIA

## Abstract

Bovine mastitis is a widespread disease in dairy cows, and is often caused by bacterial mammary gland infection. Mastitis causes reduced milk production and leads to excessive use of antibiotics. We present meta-analysis of transcriptional profiles of bovine mastitis from 10 studies and 307 microarrays, allowing identification of much larger sets of affected genes than any individual study. Combining multiple studies provides insight into the molecular effects of *Escherichia coli* infection *in vivo* and uncovers differences between the consequences of *E*. *coli* vs. *Staphylococcus aureus* infection of primary mammary epithelial cells (PMECs). In udders, live *E*. *coli* elicits inflammatory and immune defenses through numerous cytokines and chemokines. Importantly, *E*. *coli* infection causes downregulation of genes encoding lipid biosynthesis enzymes that are involved in milk production. Additionally, host metabolism is generally suppressed. Finally, defensins and bacteria-recognition genes are upregulated, while the expression of the extracellular matrix protein transcripts is silenced. In PMECs, heat-inactivated *E*. *coli* elicits expression of ribosomal, cytoskeletal and angiogenic signaling genes, and causes suppression of the cell cycle and energy production genes. We hypothesize that heat-inactivated *E*. *coli* may have prophylactic effects against mastitis. Heat-inactivated *S*. *aureus* promotes stronger inflammatory and immune defenses than *E*. *coli*. Lipopolysaccharide by itself induces MHC antigen presentation components, an effect not seen in response to *E*. *coli* bacteria. These results provide the basis for strategies to prevent and treat mastitis and may lead to the reduction in the use of antibiotics.

## Introduction

Mastitis is, arguably, the most important disease of dairy cattle [1, 2]. It is often caused by the infection of the mammary gland by various micro-organisms, including *E*. *coli*, *Streptococcus uberis* and *Staphylococcus aureus* [3–6]. Mastitis causes reduced milk production in affected cows, premature culling, discarding of inferior quality milk, veterinary and labor costs and the pervasive use of antibiotics [7].

*Escherichia coli* and *S*. *aureus* infections result in different symptoms and cellular responses. *Escherichia coli* infection is typically associated with an acute and severe form of mastitis, while *S*. *aureus* causes often a chronic but sub-clinical disease. In bovine primary mammary epithelial cells (PMECs), *E*. *coli* infection induces the expression of Toll-like receptor 2 (TLR2) and Toll-like receptor 4 (TLR4), and cytokines Tumor Necrosis Factor-α, Interleukin-1α, Interleukin-6 and Interleukin-8, and activation of the NFκB pathway; on the other hand, while *S*. *aureus* infection induces TLR2 expression, other molecular responses are delayed if present at all [8–11].

There have been significant attempts to prevent or ameliorate the consequences of bovine mastitis. For example, lipopolysaccharide (LPS) can be used to stimulate the inflammatory reactions in udders; such treatments may reduce the severity of subsequent infections [12, 13]. Lipopolysaccharide is recognized by TLR4, which may prime the innate immune system to recognize Gram-negative pathogens, such as *E*. *coli*. [14]. Mastitis is commonly treated with antibiotics [15], which has disadvantages including development of resistance and the need for increasing dosage [16].

The responses to mastitis infection have been studied using transcriptional profiling, both in infected udders *in vivo*, as well as by treating PMECs with heat-inactivated bacteria *in vitro* [17–23]. Drawing conclusions from these studies is hindered by extensive differences in individual responses between cows, even when the cows came from the same herd, with similar genetic backgrounds and similar age [24]. Recently, important gene-wide association studies between DNA polymorphisms and mastitis susceptibility in dairy cows, and these have been correlated with changes in gene expression [25–27]. While, in the same animal, responses are similar between repeated infections [28], different animals will respond inconsistently to *E*. *coli* infection [29–31]. Combining data from many studies using meta-analysis can bypass the challenges associated with individual variations, and addresses a much larger set of comparisons than any individual study [32, 33].

Here we assemble and present a meta-analysis comprising 307 microarrays from 10 individual studies of mastitis-related transcriptional profiling of responses to *E*. *coli* and *S*. *aureus*. Combining multiple studies, we were able to identify large sets of differentially regulated genes, which allowed us insights into the molecular effects of *E*. *coli* infection *in vivo*. Additionally, we found differences between *E*. *coli* and *S*. *aureus* infections of PMECs. We found that lipid biosynthesis enzymes involved in milk production are repressed under *E*. *coli* infection, which provides molecular insight into reduced milk production in infected animals. We defined the specific effects of heat-treated *E*. *coli in vitro*, which, we propose, may have prophylactic effects against mastitis. We also identify responses to bacterial LPS that are not elicited by live bacteria. The results provide insight for developing strategies to prevent and treat mastitis and may lead to the reduction in the use of antibiotics in its treatment.

## Methods

### Downloading the data files

Searching GEO Datasets for the key term “*mastitis*” and selecting “*Bos taurus*” as the organism yielded twenty nine data sets as output. From these, we selected studies focused on responses of the epithelial cells to a mastitis-causing bacterium, *E*. *coli or S*. *aureus*, either conducted *in vivo* (udder tissue) or *in vitro* (mammary epithelial cells). We did not analyze systemic responses in blood cells. The selected studies used the “Affymetrix Bovine Genome Array” platform containing 24128 genes. Additional studies were found using non-Affymetrix microarrays, but we decided not to include these for the following reasons: 1. such studies mostly used in-house microarrays, which incompletely overlap the Affymetrix arrays, and therefore would significantly reduce the total number of genes studied; 2. Each of the in-house array is used in just a few datasets (at most 3 datasets, e.g., for GPL8776, or GPL6082); 3. They used two-color RNA labeling approach, which yields relative expression values, which are not easily integrated with the Affymetrix studies; 4. The Affymetrix studies can analyze a high number of samples, and employ standardized quality controls and analysis algorithms, which can be used across different studies. The.CEL or.TXT files deposited from these studies were downloaded and unzipped, then log_2_ transformed. Datasets obtained were combined and analyzed using RMAExpress for quality control [33, 34]. For each study, data obtained from bacteria-treated and untreated, control cells were saved in different columns of Excel spread sheets (Table 1).

**Table 1:** Studies details.

No	Acc. No	Total M.A.	M.A. C+T	Bacterial strain	Tissue or Cell type	Treatment time (h)
**Live *Escerichia coli***						
**1**	GSE15020	10	5+5	*E*. *coli* 1303	Udder biopsy	24
**2**	GSE15019	10	5+5	*E*. *coli* 1303	Udder biopsy	6
**3**	GSE24217	49	23+26	*E*. *coli* k2bh2	Udder biopsy	24, 192
**4**	GSE50685	20	5+15	*E*. *coli* ECC-Z	Udder biopsy	24, 48
**Heat-Inactivated *Escerichia coli***						
**5**	GSE24560	58	27+31	*E*. *coli* 1303	PMEC	1, 6, 24
**6**	GSE25413	18	6+12	*E*. *coli* 1303	PMEC	1, 3, 6, 24
**7**	GSE32186	12	6+6	*E*. *coli* 1303	PMEC	6
**Heat-Inactivated *Staphylococcus aureus***						
**8**	GSE24560	57	27+30	*S*. *aureus* M60	PMEC	1, 6, 24
**9**	GSE25413	18	6+12	*S*. *aureus* 1027	PMEC	1, 3, 6, 24
**Lipopolysaccharide**						
**10**	GSE32186	12	6+6	LPS	PMEC	6

M.A. C+T stands for number of microarrays, control (C) and treated (T); PMEC for primary mammary epithelial cells; Acc. No. for accession number.

### Grouping studies for analysis using RankProd software

For global comparison of the expression profiles of *E*. *coli*-treated and control samples, we combined microarray data containing the 177 microarrays from the *E*. *coli* experiments into a single spreadsheet, using data-loader. We performed four separate analyses: 1) 4 studies comprising 89 microarrays for control and *E*. *coli*-infected udder biopsies. Differentially expressed genes in each of the class were recorded [21–23]. 2) Data of heat-inactivated *E*. *coli*-treated PMEC containing three data sets with 49 treated samples and 39 controls [18–20]. 3) Microarray data for LPS-treated and untreated samples from one study with 12 microarrays [18]. 4) Two studies with 75 microarrays from treated and control samples for PMEC responses to heat-inactivated *S*. *aureus* [19, 20]. Several strains of *E*. *coli* and *S*. *aureus* were used in these studies, specifically, *E*. *coli* 1303, *E*. *coli* k2bh2, *E*. *coli* ECC-Z, *S*. *aureus* M60 and *S*. *aureus* 1027 (Table 1). The animals used in these studies are from three different countries, Germany (GSE15020, GSE15019), Denmark (GSE24217) and the USA (GSE50685).

**Fig 1:**
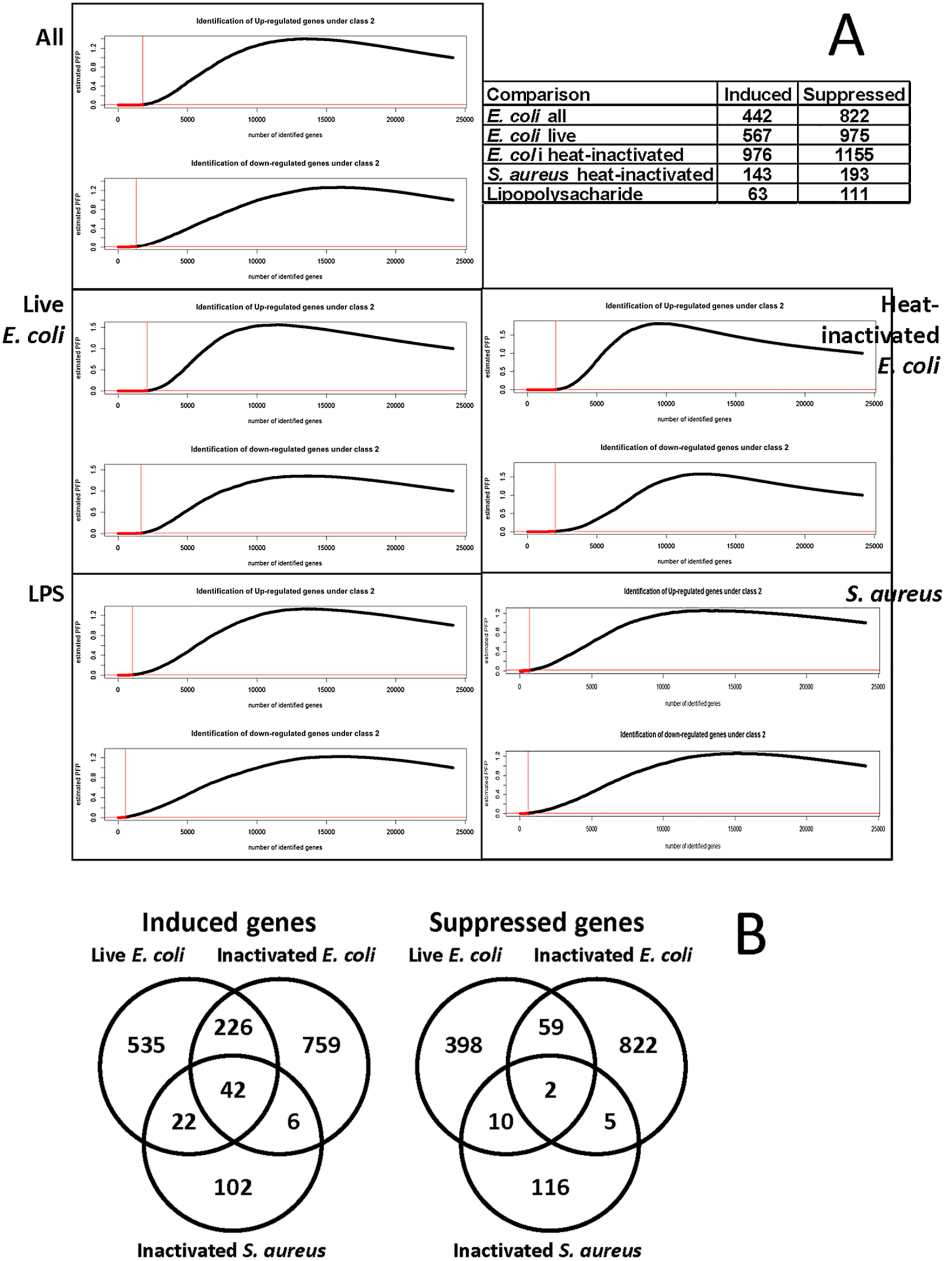
Selection of regulated genes using nonparametric RankProd evaluation. A) The genes differentially expressed with a p-value better than 0.01 are marked with dashed line. The table inset shows the numbers of regulated genes used in analysis, selected with a 10^−4^ cut-off, except for the LPS treatment, where we used 10^−3^ cut-off because a single study provided statistically less significant values. B) Venn diagrams of overlaps among the selected genes. Note that the more extensive overlaps between the *E*. *coli* regulated genes may be due to the larger numbers of such genes, when compared to the list of genes regulated by *S*. *aureus*. For studies used in this figure please refer to Table 1.

We used the RankProd Software to identify the differentially expressed genes with p-values better than 10^−4^, when compared with respective controls in the following data sets: global, live *E*. *coli-*, heat-inactivated *E*. *coli-* and heat-inactivated *S*. *aureus*-treated samples. For each analysis, the number of genes induced or suppressed in the respective comparison is recorded in Fig 1.

### Ontological Analysis

We chose genes with p-values better than our threshold from RankProd output and used online Database for Annotation, Visualization and Integrated Discovery (DAVID) software for further analysis as described before [33, 35]. For differentially expressed genes in the LPS-treated and control PMEC, we chose those with p-values better than 10^−3^. We also generated clusters of ontological categories containing extensively overlapping sets of genes, which condensed some redundancies in the regulated ontological categories. We separately identified ontological data for the induced and suppressed ontological clusters and genes in each comparison.

The PRISMA Checklist is included as S1 PRISMA Checklist.

## Results

### Datasets characterization

We searched GEO DataSets using key terms “*mastitis*” and “*Bos taurus*” and selected studies using Affymetrix bovine microarrays platform only. We found that studies describing transcriptional responses to live *E*. *coli* strains were conducted *in vivo* in udder tissues, while the responses to heat-inactivated *E*. *coli*, *S*. *aureus* or LPS were studied in primary cultures of mammary epithelial cells. We analyzed the gene ontologies upregulated and downregulated in these data sets separately (Table 1). We found ten appropriate studies containing 307 microarrays. In four studies, live *E*. *coli* were used *in vivo*, in three heat-inactivated *E*. *coli* was used on PMEC *in vitro*, in two studies similarly heat-inactivated *S*. *aureus* was used and we found a single study using LPS.

### The effects of live *E*. *coli*

The most prominent cluster of ontological categories induced by live *E*. *coli* comprises wound responses, defense and inflammatory responses, Table 2. The defense genes induced are listed in Table 3. Highly prominent in the list are genes encoding CCL and CXCL chemokines, the secreted polypeptides mediating chemotactic signals that attract macrophages, mast cells, eosinophils and neutrophils. Additional genes encoding proinflammatory polypeptides, such as IL-1α, IL-1β and vanin, are also induced. The taxis cluster, the third most prominent cluster induced by *E*. *coli* (Table 2), is an element of the wound response. It comprises the set of chemokines listed in Table 3. Similarly, vasculature development/angiogenesis is prominent in the induced categories. We also note the abundant presence of complement components. Importantly, defensins, which can be produced by the epithelia and are directly bactericidal or bacteriostatic, are strongly induced by live *E*. *coli*; these include beta-defensins DEFB10, DEFB4A, BNBD-9, as well as defensin genes LAP, LBP, LTF, and LYZ2. Live *E*. *coli* infection also upregulates expression of additional constituents of the innate responses, including CD14, TLR2 and PYCARD, proteins that recognize and orchestrate responses to bacterial infection.

**Table 2:** Clusters of ontological categories suppressed or induced by *E*. *coli* infection in cow udders *in vivo*.

	INDUCED Ontological categories	p Value		SUPRESSED Ontological categories	p Value
**1**	**14.88**		**1**	**3.81**	
	response to wounding	1.59E-16		polysaccharide binding	6.77E-05
	defense response	2.46E-15		glycosaminoglycan binding	9.94E-05
	inflammatory response	5.99E-15	**2**	**3.78**	
**2**	**11.01**			carboxylic acid biosynthetic process	7.16E-05
	extracellular region	2.40E-12		lipid biosynthetic process	7.53E-05
	extracellular space	1.16E-11	**3**	**2.99**	
**3**	**5.60**			extracellular region part	1.01E-04
	taxis	6.88E-09		extracellular matrix	6.04E-04
	chemokine receptor binding	6.12E-06	**4**	**2.31**	
**4**	**4.82**			glucose transport	3.03E-03
	lysosome	1.73E-06		hexose transport	3.96E-03
	lytic vacuole	1.73E-06	**5**	**2.09**	
**5**	**4.61**			skeletal system development	5.22E-04
	protein dimerization activity	1.21E-06		ossification	3.80E-03
	identical protein binding	6.24E-06	**6**	**1.91**	
**6**	**4.59**			aromatic compound catabolic process	3.03E-03
	vasculature development	4.64E-06		L-phenylalanine metabolic process	7.39E-03
	blood vessel development	1.19E-05	**7**	**1.85**	
**7**	**3.92**			gland development	4.56E-03
	carbohydrate binding	8.70E-06		mammary gland development	1.99E-02
	glycosaminoglycan binding	1.30E-04	**8**	**1.53**	
**8**	**3.78**			isoprenoid metabolic process	8.22E-03
	melanosome	4.61E-05		Cholesterol biosynthesis	2.85E-02
	cytoplasmic vesicle	3.53E-04	**9**	**1.46**	
**9**	**3.66**			tissue morphogenesis	2.43E-02
	endocytosis	1.11E-05		epidermis morphogenesis	2.47E-02
	phagocytosis	9.82E-04		serine/threonine kinase signaling	2.92E-02
**10**	**3.38**		**10**	**1.44**	
	negative regulation of apoptosis	7.46E-06		Viral myocarditis	6.94E-03
	anti-apoptosis	5.50E-03		MHC class II protein complex	1.62E-02

The top ten clusters with best enrichment scores are shown. The p-values are noted for individual ontological categories in each cluster.

**Table 3:** Defense response genes induced in udder *in vivo* by *E*. *coli*.

Symbol	Name	Function
**BCL2**	B-cell CLL/lymphoma 2	Transcription
**BNBD-9-LIKE**	BNBD-9-LIKE	Bactericidal activity
**C1S**	complement component 1, s	Peptidase
**C3**	complement component 3	Complement activation
**C4BPA**	complement component 4 bp, alpha	Complement activation
**C6**	complement component 6	Lytic complex formation
**CCL20**	chemokine (C-C motif) ligand 20	Chemotactic factor
**CCL3**	chemokine (C-C motif) ligand 3	inflammation and chemokine
**CCL4**	chemokine (C-C motif) ligand 4	inflammation and chemokine
**CCL5**	chemokine (C-C motif) ligand 5	Chemotactic factor
**CCR5**	chemokine (C-C motif) receptor 5	Chemokine Receptor
**CD14**	CD14 molecule	Mediates response to LPS
**CFB**	complement factor B	Complement component cleavage
**COTL1**	coactosin-like 1 (Dictyostelium)	Binding to F-actin
**CXCL11**	chemokine (C-X-C motif) ligand 11	Chemotactic factor
**CXCL16**	chemokine (C-X-C motif) ligand 16	Chemotactic response
**CYBA**	cytochrome b-245 alpha	Critical in Phagocyte oxidation
**DEFB10**	beta-defensin 10	Bactericidal activity
**DEFB4A**	beta-defensin 4	Bactericidal activity
**FCER1G**	Fc fragment of IgE	Immune response regulation
**FGR**	Gardner-Rasheed feline	Catalysis
**FN1**	fibronectin 1	Cell surface and compounds binding
**HMOX1**	heme oxygenase (decycling) 1	catalysis
**IL1A**	interleukin 1, alpha	Stimulate thymocyte proliferation
**IL1B**	interleukin 1, beta	Stimulate thymocyte proliferation
**ITGB6**	integrin, beta 6	Receptor for fibronectin and cytoactin
**LAP**	lingual antimicrobial peptide	Antibacterial and antifungal activities
**LBP**	lipopolysaccharide binding protein	Bactericidal activity
**LOC504773**	regakine 1	Immunoattractant
**LTF**	lactotransferrin	catalytic activity
**LYZ2**	lysozyme C-2	catalytic activity
**NCF1**	neutrophil cytosolic factor 1	NADPH activation
**NFKBIZ**	NF kappa B-cells inhibitor zeta	NFkB signaling
**NOS2**	nitric oxide synthase 2	catalytic activity
**OLR1**	oxidized LDL receptor 1	Involved in degradation of oLDL
**ORM1**	alpha-1 acid glycoprotein	Modulate immune system activity
**PTAFR**	platelet-activating factor receptor	inflammation
**PYCARD**	PYD and CARD domain containing	Promotes caspase-mediated apoptosis
**RAB27A**	member RAS oncogene family	GTPase superfamily
**S100A12**	S100 calcium binding protein A12	Belongs to the S-100 family
**SAA3**	serum amyloid A3	Major acute phase reactant
**SELP**	selectin P	Receptor for myeloid cells
**SERPINF2**	serpin peptidase inhibitor	Plasmin, trypsin, chymotrypsin inhibitor
**THBS1**	thrombospondin 1	Cell to cell or matrix interaction mediator
**TLR2**	toll-like receptor 2	Mediates response to LPS
**VNN1**	vanin 1	catalytic activity

The second most prominent induced cluster comprises genes encoding extracellular proteins (Table 2). The character of the secreted proteins in the induced and suppressed sets is diametrically different: while genes encoding small signaling polypeptides, growth factors, cytokines and chemokines are induced (Table 4A), the much larger basement membrane, extracellular matrix and cell attachment protein genes are suppressed (Table 4B). Essentially, *E*. *coli*-infected epithelia express secreted proinflammatory signals and concomitantly relax their attachment to the dermal connective tissue.

**Table 4:** Genes encoding extracellular proteins.

**Table 4A: Extracellular Region Genes INDUCED by *E*. *coli***			
**Symbol**	**Name**	**Function**	
**ADM**	adrenomedullin	Hypotensive peptide controls circulation	**Signaling**
**ALB**	albumin	allergic reaction in human	
**ANGPT2**	angiopoietin 2	counteracts blood vessel maturation	**Signaling**
**ANGPTL4**	angiopoietin-like 4	hypoxia-induced expression in endothelial cells	**Signaling**
**APOE**	apolipoprotein E	Mediates the binding, internalization, and catabolism of LPS	**Signaling**
**C3**	complement 3	Complement activation	**Signaling**
**CALR**	calreticulin	interacts with monoglucosylated proteins synthesized in ER	
**CCL19**	chemokine (C-C) 19	inflammatory and immunological responses	**Signaling**
**CCL2**	chemokine (C-C) 2	Chemoattractant for monocytes	**Signaling**
**CCL20**	chemokine (C-C) 20	Chemoattractant for lymphocytes and neutrophils	**Signaling**
**CCL3**	chemokine (C-C) 3	inflammatory and chemokinetic properties	**Signaling**
**CCL4**	chemokine (C-C) 4	inflammatory and chemokinetic properties	**Signaling**
**CCL5**	chemokine (C-C) 5	Chemoattractant for monocytes, T-helper cells and eosinophils	**Signaling**
**CHI3L1**	chitinase 3-like 1	defense against pathogens or in tissue remodeling	**Signaling**
**COL1A2**	collagen I, alpha 2	fibrillar forming collagen	**ECM**
**CXCL11**	chemokine (C-X-C) 11	Chemotactic for IL-activated T-cells	**Signaling**
**CXCL13**	chemokine (C-X-C) 13	Chemotactic for B-lymphocytes	**Signaling**
**CXCL16**	chemokine (C-X-C)16	Induces chemotactic response	**Signaling**
**ECM1**	extracellular matrix protein 1	promotes angiogenesis, ossification and endothelial cells prolif.	**ECM**
**EDN1**	endothelin 1	Potent vasoconstrictor	**Signaling**
**FGF1**	fibroblast growth factor 1	angiogenic agents and potent mitogens	**Signaling**
**FGL2**	fibrinogen-like 2	contributes in physiologic lymphocyte functions at mucosal sites	**ECM**
**GPX3**	glutathione peroxidase 3	Protects cells and enzymes from oxidative damage	
**HP**	haptoglobin	protects kidneys from damage by hemoglobin ICAM1	
**ICAM1**	intercellular adhesion molecule 1	ligand for leukocyte adhesion protein LFA-1	**Signaling**
**IFNAR2**	interferon receptor 2	signal transduction interacting TK-JAK1	**Signaling**
**IGFBP4**	insulin like GF binding protein 4	inhibit or stimulate growth promoting effects of IGFs	**Signaling**
**IL18**	interleukin 18	Stimulates natural killer cell activity and IFN-ɣ production	**Signaling**
**IL1A**	interleukin 1, alpha	inflammatory response	**Signaling**
**IL1B**	interleukin 1, beta	inflammatory response	**Signaling**
**IL1RN**	interleukin1 receptor antagonist	Inhibits activity of IL-1	**Signaling**
**LBP**	LPS binding protein	Binds to LPS	**Signaling**
**LGALS1**	lectin galactoside-binding soluble1	regulates apoptosis, cell proliferation and cell differentiation	**Signaling**
**LOC504773**	regakine 1	Chemotactic for neutrophils and lymphocytes	**Signaling**
**MMP9**	matrix metallopeptidase 9	Functions in bone osteoclastic resorption	**ECM**
**ORM1**	alpha-1 acid glycoprotein	modulate immune system during acute-phase reaction	**Signaling**
**PDIA3**	disulfide isomerase family A,3	Catalyzes rearrangement of -S-S- bonds in proteins	
**PLA2G7**	phospholipase A2, group VII	Modulates action of platelet activating factor	**Signaling**
**RBP4**	retinol binding protein 4	Delivers retinol from liver to peripheral tissues	**Signaling**
**SAA3**	serum amyloid A 3	acute phase reactant, Apolipoprotein of HDL complex	**Signaling**
**SERPINA1**	serpin peptidase inhibitor cladeA, 1	Inhibitor of serine proteases	**Signaling**
**SERPINA3-1**	serpin peptidase inhibitor clade A,3	inhibitor of serine proteases	**Signaling**
**SERPINF1**	serpin peptidase inhibitor clade F, 1	induces neuronal differentiation and inhibitor of angiogenesis	**Signaling**
**SRGN**	serglycin	lytic vacuole	**Signaling**
**THBS1**	thrombospondin 1	mediates cell-to-cell and cell-to-matrix interactions	**ECM**
**VEGFC**	vascular endothelial growth factor C	Belongs to the PDGF/VEGF growth factor family	**Signaling**
**Table 4B: Extracellular Region Genes SUPRESSED by *E*. *coli***			
**CCDC80**	coiled-coil domain containing 80	regulation of cell-substrate adhesion	**ECM**
**CMTM8**	CKLF-like MARVEL domain 8	cytokine activity	**Signaling**
**COL17A1**	collagen type 17 alpha 1	hemidesmosome integrity and basal keratinocytes attachment	**ECM**
**COL1A2**	collagen type I alpha 2	Focal adhesion	**ECM**
**CRISPLD2**	cysteine-rich protein LCCL domain2	Promotes matrix assembly	**ECM**
**FMOD**	fibromodulin	Affects fibrils formation rate	**ECM**
**EGFLAM**	EGF-like fibronectin typeIII & laminin G domains	Carbohydrate binding	**ECM**
**FGL1**	fibrinogen like 1	hepatocyte mitogenic activity	**ECM**
**HAPLN1**	hyaluronan and proteoglycan link protein1	Stabilizes aggregates of proteoglycan with hyaluronic acid	**ECM**
**KERA**	keratocan	functions in corneal transparency and stromal matrix structure	**ECM**
**KIT**	v-kit Hardy-Zuckerman 4	catalytic activity in oocyte growth	
**LOXL1**	lysyl oxidase like 1	Active on elastin and collagen substrates	**ECM**
**LOXL4**	lysyl oxidase like 4	modulate formation of collagenous extracellular matrix	**ECM**
**LPL**	lipoprotein lipase	catalytic activity	
**LPO**	lactoperoxidase	catalytic activity	
**LUM**	lumican	important in development of tissue engineered cartilage	**ECM**
**MFAP4**	microfibrillar associated protein 4	involved in Ca-dependent cell adhesion or intercell. interactions	**ECM**
**MFGE8**	milk fat globule-EGF factor 8	Binds to phosphatidylserine cell surfaces	
**MSR1**	macrophage scavenger receptor 1	mediate endocytosis of diverse group of macromolecules	
**MSTN**	myostatin	Cytokin and growth factor activity	**Signaling**
**MYOC**	myocilin	trabecular meshwork inducible glucocorticoid response	**ECM**
**NTN4**	netrin 4	neuron remodeling	**Signaling**
**OGN**	osteoglycin	Induces bone formation	**Signaling**
**POSTN**	periostin osteoblast specific factor	important in extracellular matrix mineralization	**ECM**
**PRELP**	proline/arginine-rich end leucine-rich repeat	anchor basement membranes to underlying connective tissue	**ECM**
**PRSS2**	protease serine, 2	catalytic activity	
**TFF3**	trefoil factor 3	Functions as motogen and maintenance and repair of intestinal muc.	**ECM**
**TGFB2**	transforming growth factor beta 2	suppressive effects on IL-2 dependent T-cell growth	**Signaling**
**THBS1**	thrombospondin 1	mediates cell-to-cell and cell-to-matrix interactions	**ECM**
**VLDLR**	very low density lipoprotein receptor	receptor-mediated endocytosis of specific ligands	**Signaling**

**A) INDUCED by *E*. *coli*. B) SUPRESSED by *E*. *coli*.** Most of the induced genes encode cytokines and related small signaling polypeptides, whereas most of the suppressed genes encode large extracellular matrix proteins. Data derive from the *in vivo* experiments.

*Escherichia coli* induces *in vivo* the expression of several types of genes encoding intracellular vesicle proteins, lysosomal, melanocytic and endo-phagocytotic (Table 2). We also note that the anti-apoptotic genes are induced in the infected tissue.

Prominent clusters comprise extracellular matrix proteins, as already described. However, particularly remarkable is the second cluster, comprising the carboxylic acid/lipid biosynthesis enzymes: of the 20 genes in this cluster, 11 are directly related to milk production (Table 5). This result clearly identifies the molecular mechanism responsible for the reduced milk production in cows affected by mastitis.

**Table 5:** Metabolic enzymes suppressed by *E*. *coli*.

Symbol	Function	
**ACACA**	sheep milk	Milk-related
**ACSM1**	Gland development	
**AGPAT1**	Milk production	Milk-related
**AGPAT6**	Milk production	Milk-related
**ALOX15**	Inflammatory responses	
**BCAT2**	Cellular a.a. catabolism	
**CBS**	Sulphur a.a. metabolism	
**COQ2**	ubiquinone biosynthesis	
**FASN**	effects milk fat content	Milk-related
**FDFT1**	Imp for Milk yield and quality	Milk-related
**GPAM**	Milk production	Milk-related
**HMGCR**	Cholestrol synthesis	
**LPL**	Present in milk	Milk-related
**LTA4H**	FA Biosynthesis	Milk-related
**MVK**	FA Biosynthesis	Milk-related
**PEMT**	required for lactation and pregnancy	Milk-related
**PSAT1**	VitB6 (comp of milk) metabolism	Milk-related
**PYCR1**	Arginine and proline metabolism	
**SCD**	biosynthesis of unsaturated FA	
**TM7SF2**	Steroid biosynthesis	

Many genes necessary for milk production are downregulated under *E*. *coli* infection. Data derive from the *in vivo* experiments.

Furthermore, *E*. *coli* infection *in vivo* suppresses several metabolic processes: glucose transport, amino acid and cholesterol metabolism, etc. In addition, *E*. *coli* infection suppresses the differentiation of epithelial cells, specifically keratinocyte differentiation. Collectively, in the epithelial cells *E*. *coli* infection compromises milk-production and homeostasis at the transcriptional level.

### The effects of heat-inactivated *E*. *coli*

We analyzed a set of experiments performed with heat-inactivated *E*. *coli* to define their effects on PMECs *in vitro* [18–20]. It is important to note that the heat-inactivated *E*. *coli* was used *in vitro*, with monocultures of PMEC, while the live *E*. *coli* was used *in vivo* in cow udders, which are complex multi-tissue organs. Therefore, we cannot, at this point, distinguish the differences due to the heat-inactivation of the bacteria from those due to the *in vivo/in vitro* dichotomy. Table 6 lists the regulated ontological categories. The most prominently induced category comprises genes encoding ribosomal proteins. Detailed study of the category shows enhanced ribosomal structural gene expression. The second most prominent category comprises genes encoding cytoskeletal proteins. In contrast to the *in vivo* results with live *E*. *coli*, a prominent upregulated ontological category is programmed cell death, which contains genes involved in positive regulation of apoptosis, namely caspases, hydrolases, peptidases and apoptotic mitochondrial genes. We found some bacterial toxin-response genes in this category as well. Similarly to the *in vivo* results, PMECs react to *E*. *coli* treatment by upregulating secreted signaling polypeptides, in particular angiogenic ones. This category includes genes contributing to cell attachment, morphogenesis and wound healing. We also found that ontological categories of “pigment granules” or “melanocytes” are significantly overrepresented; however, it is important to note that the genes present in these categories are principally heat shock proteins and chaperones, which bind to LPS of bacterial origin and initiate inflammatory response, including TNFα secretion; on the other hand, the encoded proteins may not be directly involved in melanogenesis. Transcription of the proteasome complex, containing threonine-type endopeptidases involved in protein degradation, is also increased.

**Table 6:** Top 10 Clusters of ontological categories suppressed or induced by heat-inactivated *E*. *coli*.

Table 6: Ontological Categories in PMECs Treated with Heat-Inactivated *E*. *coli*					
	INDUCED			SUPRESSED	
	Ontological categories	p Value		Ontological categories	p Value
**1**	**20.38**		**1**	**14.42**	
	Ribosome	1.84E-30		organelle inner membrane	2.51E-20
	translation	1.66E-22		Oxidative phosphorylation	1.62E-15
**2**	**11.81**		**2**	**4.23**	
	structural molecule activity	2.62E-20		vesicle	4.18E-05
	cytoskeleton	2.28E-04		melanosome	7.65E-05
**3**	**7.11**		**3**	**3.77**	
	apoptosis	2.07E-08		cell cycle	4.29E-07
	programmed cell death	3.66E-08		mitosis	6.18E-04
**4**	**5.11**		**4**	**3.72**	
	pigment granule	8.05E-08		NADH dehydrogenase activity	4.34E-05
	melanosome	8.05E-08		oxidoreductase activity	1.57E-04
**5**	**4.76**		**5**	**3.60**	
	vasculature development	7.03E-07		membrane-enclosed lumen	3.00E-07
	angiogenesis	5.44E-04		nuclear lumen	2.07E-03
**6**	**4.57**		**6**	**3.34**	
	proteasome complex	3.62E-08		extracellular structure organization	2.79E-04
	proteasome core complex, alpha-subunit complex	1.23E-02		collagen fibril organization	8.73E-04
**7**	**3.80**		**7**	**3.14**	
	extracellular region part	8.07E-06		translation factor activity, nucleic acid binding	2.70E-04
	extracellular region	2.09E-02		translation initiation factor activity	6.43E-04
**8**	**3.68**		**8**	**2.85**	
	regulation of protein kinase cascade	3.35E-05		cell-matrix adhesion	3.97E-06
	regulation of I-kappaB kinase/NF-kappaB cascade	6.59E-05		integrin binding	1.74E-04
**9**	**3.57**		**9**	**2.72**	
	positive regulation of cell motion	7.29E-05		vacuole	9.78E-04
	regulation of cell motion	7.64E-05		lytic vacuole	1.05E-03
**10**	**3.28**		**10**	**2.67**	
	regulation of apoptosis	2.78E-06		extracellular matrix part	6.25E-05
	positive regulation of programmed cell death	2.49E-04		proteinaceous extracellular matrix	1.05E-04
**14**	**2.68**				
	defense response	8.29E-04			
	inflammatory response	2.20E-03			
	response to wounding	5.11E-03			
**24**	**2.14**				
	epithelial cell differentiation	7.30E-04			
	keratinocyte differentiation	6.94E-02			
**25**	**2.12**				
	Toll-like receptor signaling pathway	9.90E-05			
	RIG-I-like receptor signaling pathway	7.95E-02			

Additional three clusters, ranked 14, 24 and 25^th^ are shown in the induced category for comparison with the data in Table 2. All these have enrichment scores better than 2. The p-values are noted for individual ontological categories in each cluster.

Inflammatory, defense, wound healing and bacterial recognition mechanisms, both the Toll-like and the RIG-like (retinoic-acid-inducible protein 1-like) receptor signaling pathways, are upregulated but less prominent in heat-inactivated *E*. *coli-*treated PMECs (Table 6B), where production of membrane-enclosed organelles and vesicles, in particular mitochondria, is suppressed. Notably, genes encoding nuclear and cell cycle proteins are also suppressed. This is distinct from the processes suppressed by live *E*. *coli in vivo*. As *in vivo*, the genes encoding extracellular matrix and basement membrane proteins are suppressed by the heat-inactivated *E*. *coli*.

Overall, the heat-inactivated *E*. *coli* regulates a different set of genes from the one regulated by live *E*. *coli*: specifically 1) the metabolic enzymes of lipid biosynthesis and sugar transport are not suppressed and 2) inflammation- and defense-related genes are much attenuated in response to heat-inactivated *E*. *coli*.

### The effects of *S*. *aureus*

Infections with *S*. *aureus* tend to be milder and cause less significant mastitis morbidity than those with *E*. *coli* [3, 7]. Several studies reported the transcriptional profiles of heat-inactivated *S*. *aureus* treatment of PMECs [9, 10, 19, 20]. These are directly comparable with the profiles of *E*. *coli*-treated PMECs shown above. In the *S*. *aureus* treated PMECs, the most prominently induced cluster comprises inflammatory, immune and defense responses (Table 7). Heat-inactivated *S*. *aureus* is much more proficient in eliciting these responses than is *E*. *coli*. The defense responses include extracellular signaling peptides, cell adhesion molecules, inducers of acute inflammation, regulators of lymphocyte-mediated immunity, etc. We also note quite prominent induction of receptors responsible for recognition of microbes by innate immunity, namely NOD- and Toll-like receptors.

**Table 7:** Clusters of ontological categories suppressed or induced by *S*. *aureus*.

Table 7: Ontological Categories in PMECs Treated with Heat-Inactivated *S*. *aureus*					
	INDUCED			SUPRESSED	
	Ontological categories	P-Value		Ontological categories	P-Value
**1**	**10.70**		**1**	**4.33**	
	inflammatory response	9.84E-14		cell migration	2.36E-05
	defense response	6.59E-13		localization of cell	4.06E-05
	immune response	1.01E-12		Cell Motility	4.06E-05
**2**	**6.54**		**2**	**2.27**	
	extracellular space	5.94E-08		extracellular space	2.12E-03
	extracellular region	1.24E-07		extracellular region	1.98E-02
**3**	**4.46**		**3**	**2.24**	
	acute inflammatory response	2.23E-07		plasma membrane	2.72E-05
	positive regulation of cell component organization	3.54E-04		plasma membrane part	8.98E-05
**4**	**2.87**		**4**	**2.09**	
	Graft-versus-host disease	6.01E-06		striated muscle tissue development	1.48E-03
	Cell adhesion molecules (CAMs)	5.94E-03		striated muscle cell differentiation	5.55E-02
**5**	**2.61**		**5**	**1.71**	
	positive regulation of immune system process	8.80E-08		receptor tyrosine kinase signaling	6.68E-05
	positive regulation of cell proliferation	4.73E-03		response to peptide hormone stimulus	1.91E-02
**6**	**2.34**		**6**	**1.64**	
	acute inflammatory response	2.23E-07		receptor complex	1.27E-02
	positive regulation of response to stimulus	1.58E-04		integral to plasma membrane	2.85E-02
**7**	**2.12**		**7**	**1.45**	
	Graft-versus-host disease	6.01E-06		Focal adhesion	1.30E-03
	positive regulation of developmental process	1.03E-03		cell junction assembly	3.01E-03
**8**	**2.11**		**8**	**1.44**	
	NOD-like receptor signaling pathway	1.27E-03		tissue homeostasis	2.17E-02
	response to bacterium	2.03E-03		multicellular organismal homeostasis	3.71E-02
**9**	**1.87**		**9**	**1.39**	
	skeletal system development	7.64E-03		enzyme linked receptor signaling	8.86E-07
	ossification	1.77E-02		growth factor binding	6.41E-03
**10**	**1.51**		**10**	**1.37**	
	regulation of immune effector process	1.36E-02		MHC protein complex	1.68E-02
	regulation of lymphocyte mediated immunity	4.22E-02		antigen processing and presentation	2.45E-02
**11**	**1.48**				
	positive regulation of response to stimulus	1.58E-04			
	Toll-like receptor signaling pathway	1.81E-04			

The top 10 and top 11 clusters are given for the suppressed and induced genes, respectively.

The most conspicuous ontological categories suppressed by *S*. *aureus* involve cell migration (Table 7). Relatedly, genes encoding extracellular matrix proteins and focal adhesion components are suppressed. Proteins embedded in the plasma membrane, including growth factor-binding receptor tyrosine kinases, are also prominent.

On the whole, the transcriptional responses to *S*. *aureus* differ from those to *E*. *coli* by a significantly stronger induction of proinflammatory and immunomodulatory genes, and stronger suppression of cell attachment and motility genes. At the same time, *S*. *aureus* does not suppress the metabolic and milk lipid producing enzymes that *E*. *coli* does.

#### The effects of LPS

While *S*. *aureus* is Gram-positive, *E*. *coli* is Gram-negative and thus *E*. *coli* produces copious amounts of lipopolysaccharide, LPS. In epithelial and other cells, LPS is recognized by TLR4, which initiates a series of responses to infections with Gram-negative bacteria [14]. We hypothesized that treating PMECs with LPS would cause a subset of transcriptional responses caused by *E*. *coli*. We found a single study that treats PMECs with LPS [18] and consequently the statistical significance of the regulated genes is markedly reduced (Table 8). Nevertheless, we find that LPS treatment induces immune, inflammatory and defense response in PMECs, including the antigen processing machinery (Table 8). Proteolysis is also induced by LPS. Interestingly, apoptosis related genes seem to be induced. Very few ontological categories suppressed by LPS reached statistical significance, but we note that the genes encoding extracellular matrix proteins seem suppressed.

**Table 8:** Clusters of ontological categories suppressed or induced by LPS.

Table 8: Ontological Categories in PMECs Challenged with Lipopolysaccharide					
	INDUCED			SUPRESSED	
	Ontological categories	p Value		Ontological categories	p Value
**1**	**2.59**		**1**	**1.52**	
**#**	immune response	2.78E-08	**#**	extracellular region	1.31E-02
	positive regulation of immune system process	5.27E-03		extracellular region part	1.91E-02
**2**	**2.55**		**2**	**1.48**	
**#**	Antigen processing and presentation	3.05E-05		calcium ion binding	8.55E-03
	peptide or polysaccharide antigen via MHC class II	3.62E-03		metal ion binding	4.75E-02
**3**	**2.35**				
**#**	defense response	2.19E-06			
	immune effector process	3.29E-05			
**4**	**2.02**				
**#**	extracellular region	1.63E-03			
	inflammatory response	2.24E-03			
**5**	**1.83**				
**#**	positive regulation of endocytosis	2.10E-03			
	regulation of vesicle-mediated transport	1.83E-02			
**6**	**1.56**				
	ISG15-protein conjugation	5.72E-07			
	proteolysis	1.52E-02			
**7**	**1.25**				
	serine-type peptidase activity	2.61E-02			
	peptidase activity, acting on L-amino acid peptides	4.39E-02			
**8**	**1.03**				
	apoptosis	7.89E-02			
	programmed cell death	8.28E-02			

Only clusters with enrichment scores better than 1.0 are given. Note the significantly higher p-values due to a smaller set of microarrays analyzed. The subset of clusters regulated similarly by *E*. *coli* is marked with # signs.

We looked specifically at the set of LPS-induced genes involved in defense and immunity (Table 9). We find that many of these (6 out of 11) are components of the complement system and anti-bacterial defense genes also induced by live *E*. *coli* (cf. Table 4A). Of the LPS-induced genes not induced by live *E*. *coli*, the majority are involved in MHC antigen presentation process (Table 9). It is of interest that LPS has been proposed as a potential preventive treatment for *E*. *coli*-caused mastitis [36]. One potential mechanism may include boosting the antigen presentation machinery, which does not occur after infection with live *E*. *coli*.

**Table 9:** Defense and immunity Genes induced in LPS-challenged PMECs.

Symbol	Name	Function	
**CCL5**	chemokine (C-C motif) ligand 5	Chemotactic factor	**#**
**C2**	complement component 2	Catalytic activity	**MHC**
**C3**	complement component 3	Complement activation	**#**
**CFB**	complement factor B	Complement component cleavage	**#**
**LTF**	lactotransferrin	Catalytic activity	**#**
**LAP**	lingual antimicrobial peptide	Antibacterial and antifungal activities	**#**
**BOLA-RDA**	MHC II, DR alpha	Antigen prsentation via MHC II	**MHC**
**PTX3**	pentraxin related gene	Regulates innate resistance to pathogens	
**SAA3**	serum amyloid A3	Major acute phase reactant	**#**
**RSAD2**	radical S-adenosyl methionine domain 2	Involved in antiviral defense	
**TAP1**	transporter 1	Peptide transmembrane transport	**MHC**

The genes also induced by live *E*. *coli* (Table 3) are marked with #. Note the abundance of MHC-related genes among those NOT induced by *E*. *coli*.

Overall, these results support our hypothesis that the effects of LPS generally represent a subset of the effects of *E*. *coli*. This subset is marked with a number sign in Table 8.

## Discussion

The results presented in this work attest to the power of meta-analysis: the highly variable individual responses to mastitis bacteria could be overcome by assembling multiple analyses and thus increasing the studied population. Importantly, meta-analysis confirmed the most important findings in individual studies, namely response to wounding, inflammatory and defense responses [17–23]. Moreover, this meta-analysis provided many additional details, for example by identifying the cytokines and additional secreted signaling polypeptides produced.

Perhaps the most important novel finding from this meta-analysis concerns the specific suppression of milk-producing metabolic enzymes (Table 5). The infection would be expected to slow down anabolic processes in most cases, as the tissue has to divert energy to fighting infection. However, the unique aspect of this slow-down in bovine mastitis is reduction of milk fat production. The seven marked enzymes in Table 5 are those that are directly and specifically devoted to milk production. It is quite likely that additional enzymes, e.g., those for amino acid biosynthesis, also play important role in milk production.

Additional novel ontological categories shown to be induced in mastitis include cellular taxis, cytoplasmic vesicles and anti-apoptosis agents. Cellular taxis is predominantly related to the leucocyte infiltrates caused by copious production of chemokines and cytokines; at present we cannot exclude enhanced taxis of epithelial cells as well, which will have to be examined with laboratory-based, as well as in-the-field experiments. The vesicle-associated proteins include those related to lysosomes, endocytosis and even melanosomes. The affected cell types are probably diverse, although it should be noted that genes encoding melanosomal proteins are also induced in the primary mammary epithelial cells.

Conversely, mastitis suppresses several aspects of basic epithelial biology, including extracellular matrix biosynthesis, mammary gland development markers and epidermis morphogenesis, including cholesterol biosynthesis, an integral component of epidermal differentiation [37]. Importantly, however, the seven milk production-related enzymes mentioned above are not integral to epidermal differentiation and thus represent a specific metabolic category suppressed in mastitis.

The effects of heat-inactivated *E*. *coli* on mammary epithelial cells *in vitro* are quite different from the *in vivo* effects. For example, the inflammatory response, and cytotaxis are much attenuated; these are, presumably, induced *in vivo* in the leucocyte compartment, and so are missing from pure cultures of mammary epithelial cells. We do see induction of melanosomal genes, vesicles specific for the epidermal tissue. In these cells, apoptosis is induced as a defensive mechanism. Interestingly, the innate immunity response, an important function of keratinocytes, is induced; this includes the NFκB pathway as well as the Toll-like and RIG-like receptor signaling pathways. Importantly, heat-inactivated *E*. *coli* seem not to suppress the transcription of metabolic enzymes, including those involved in production of milk lipids.

These results lead us to suggest that the treatment of cow udders with heat-inactivated *E*. *coli* may have a prophylactic effect against mastitis. While development of vaccines to achieve acquired immunity to mastitis in cattle, though challenging, is progressing [7, 38, 39], the approaches that target the innate immunity may also prove promising. The heat-inactivated *E*. *coli* could activate the innate immunity responses with attenuated inflammatory responses, thus priming the tissue to fight subsequent infection, without the concomitant damage due to inflammation. Treatment with heat-inactivated *E*. *coli*, if effective, would have major benefits in avoiding widespread use of antibiotics, reducing the costs of treatment and, notably, fighting mastitis in the third world. In underdeveloped areas, where the use of antibiotics is unavailable or prohibitively expensive, heat-inactivation treatments could be properly and easily performed locally.

A related approach using endotoxin to elicit a mild form of mastitis in hope of avoiding subsequent infections had a limited success [13]. The lipopolysaccharide treatment of mammary epithelial cells induced immune response genes, particularly those related to the acquired immunity, including antigen processing by keratinocytes. This is very different from the responses to heat-inactivated *E*. *coli* bacteria.

As noted before, we see significant differences in responses to *E*. *coli vs*. *S*. *aureus* [9, 10, 19, 20]. While both cause robust proinflammatory and immune responses, *S*. *aureus* also induces Toll-like and NOD-like innate immunity in mammary epithelia, while suppressing cell motility, antigen presentation and receptor signaling in general, hallmarks of acquired immunity responses. These differences may account for comparatively much milder and sub-acute sequelae of *S*. *aureus*-triggered mastitis.

*Escherichia coli* and *S*. *aureus* are not the only bacterial species important in causing mastitis; our study did not include significant microarray studies with *Streptococcus uberis* [5, 6] because of limited compatibility of GPL8776 microarrays with the Affymetrix platform. However, we want to emphasize that these studies identified important differences between cows fed *ad libitum* and those with negative energy balance, showing increased expression of lipid metabolism genes in underfed cows [5, 6].

We must emphasize several caveats of our meta-analysis. Given the very individual responses in cows [24, 40–42], our ‘forest’ view may be inapplicable to ‘trees’. Second, there are two important distinctions between our largest data sets: one uses live *E*. *coli in vivo*, the other heat-inactivated *E*. *coli* on cultured cells. We cannot, from this perspective, distinguish the *in vivo/in vitro* from the live/heat-inactivated dichotomies, especially as the *in vivo* studies include mixed populations of cells in their microarrays, while the *in vitro* studies use pure populations. Third, the LPS-responsive study is compromised by its relatively small size. Fourth, all original data are obtained in western academic settings; this may inadequately represent the conditions in the field, especially in less developed agricultural areas. And fifth, in this meta-analysis we have grouped expression data from short-term, 1–3 hrs., to long-term, 8 day treatments (Table 1); we realize that mastitis-causing infections are dynamic processes and that much additional data needs to be generated before any claims regarding the course of mastitis infection can be described in detail.

Nevertheless, the meta-analysis based on large amount of original data represents an important contribution to our understanding of bovine mastitis in various aspects and provides a solid foundation for the development of new treatments for mastitis.

## Supporting Information

S1 PRISMA Checklist Supplemental information comprises the PRISMA check list only. (DOC)
