# Supplementary material for: Meta-Analysis of Transcriptional Responses to Mastitis-Causing Escherichia coli
Source: PLoS One. 2016 Mar 2;11(3):e0148562. doi: 10.1371/journal.pone.0148562 (PMC4775050; doi:10.1371/journal.pone.0148562)
Supplement: S1 PRISMA Checklist — (DOC) [file pone.0148562.s001.doc]

| **Section/topic** | **#** | **Checklist item** | **Reported on page #** |
| --- | --- | --- | --- |
| **TITLE** | | |  |
| Title | 1 | Metaanalysis of Molecular Responses to Mastitis-Causing Bacteria. | 1 |
| **ABSTRACT** | | |  |
| Structured summary | 2 | **Note: This is NOT a metaanalysis of clinical or preclinical trials**.  Some items in this list will not be applicable. This is a metaanalysis of data in microarray transcriptional profiling studies as available in public repositories. | 2 |
| **INTRODUCTION** | | |  |
| Rationale | 3 | Metaanalysis provides much more relevant data that do single transcriptomic studies. | 2 |
| Objectives | 4 | PICOS Participants: Individual cows and bovine mammary epithelial cell cultures. Interventions: None. Comparisons: Bacteria-infected vs. controls. Outcomes: Not applicable. Study design: mRNA analysis using Affymetrix microarrays. | 6,7 |
| **METHODS** | | |  |
| Protocol and registration | 5 | No review protocol | N/A |
| Eligibility criteria | 6 | Not applicable | N/A |
| Information sources | 7 | NCBI GEO datasets | 6,7 |
| Search | 8 | Keywords: Bos Taurus AND E. coli OR S. aureus | 6 |
| Study selection | 9 | Bacteria-infected vs. controls | 7 |
| Data collection process | 10 | Downloading CEL and TXT files. | 6,7 |
| Data items | 11 | Not applicable | N/A |
| Risk of bias in individual studies | 12 | Not applicable | N/A |
| Summary measures | 13 | Not applicable | N/A |
| Synthesis of results | 14 | Not applicable | N/A |

Page 1 of 2

| **Section/topic** | **#** | **Checklist item** | **Reported on page #** |
| --- | --- | --- | --- |
| Risk of bias across studies | 15 | Not applicable | N/A |
| Additional analyses | 16 | None | N/A |
| **RESULTS** | | |  |
| Study selection | 17 | 10 studies screened, assessed for eligibility, and included in the review, see flow diagram. | 7 |
| Study characteristics | 18 | All microarray data were used in their totality. | 6,7,8 |
| Risk of bias within studies | 19 | Not applicable | N/A |
| Results of individual studies | 20 | Not applicable | N/A |
| Synthesis of results | 21 | Not applicable | N/A |
| Risk of bias across studies | 22 | Not applicable | N/A |
| Additional analysis | 23 | None | N/A |
| **DISCUSSION** | | |  |
| Summary of evidence | 24 | In udders, **live** *E. coli* **elicits inflammatory and immune defenses through** numerous cytokines and chemokines. Importantly, we found downregulation of lipid biosynthesis enzymes that are involved in milk production under *E. coli* infection. Additionally, metabolism is generally suppressed. Finally, defensins and bacteria-recognition proteins are upregulated, while extracellular matrix expression is silenced. Relevant to farmers and veterinarians. | 23 |
| Limitations | 25 | None obvious. | N/A |
| Conclusions | 26 | The results provide the basis for strategies to prevent and treat mastitis and may lead to the reduction in the use of antibiotics in agriculture. | 24 |
| **FUNDING** | | |  |
| Funding | 27 | None. | N/A |

*From:*  Moher D, Liberati A, Tetzlaff J, Altman DG, The PRISMA Group (2009). Preferred Reporting Items for Systematic Reviews and Meta-Analyses: The PRISMA Statement. PLoS Med 6(6): e1000097. doi:10.1371/journal.pmed1000097

For more information, visit: **www.prisma-statement.org**.

Page 2 of 2
